# Supplementary material for: PIM1 phosphorylation of the androgen receptor and 14-3-3 ζ regulates gene transcription in prostate cancer
Source: Commun Biol. 2021 Oct 25;4:1221. doi: 10.1038/s42003-021-02723-9 (PMC8546101; doi:10.1038/s42003-021-02723-9)

Supplementary Figure 1.

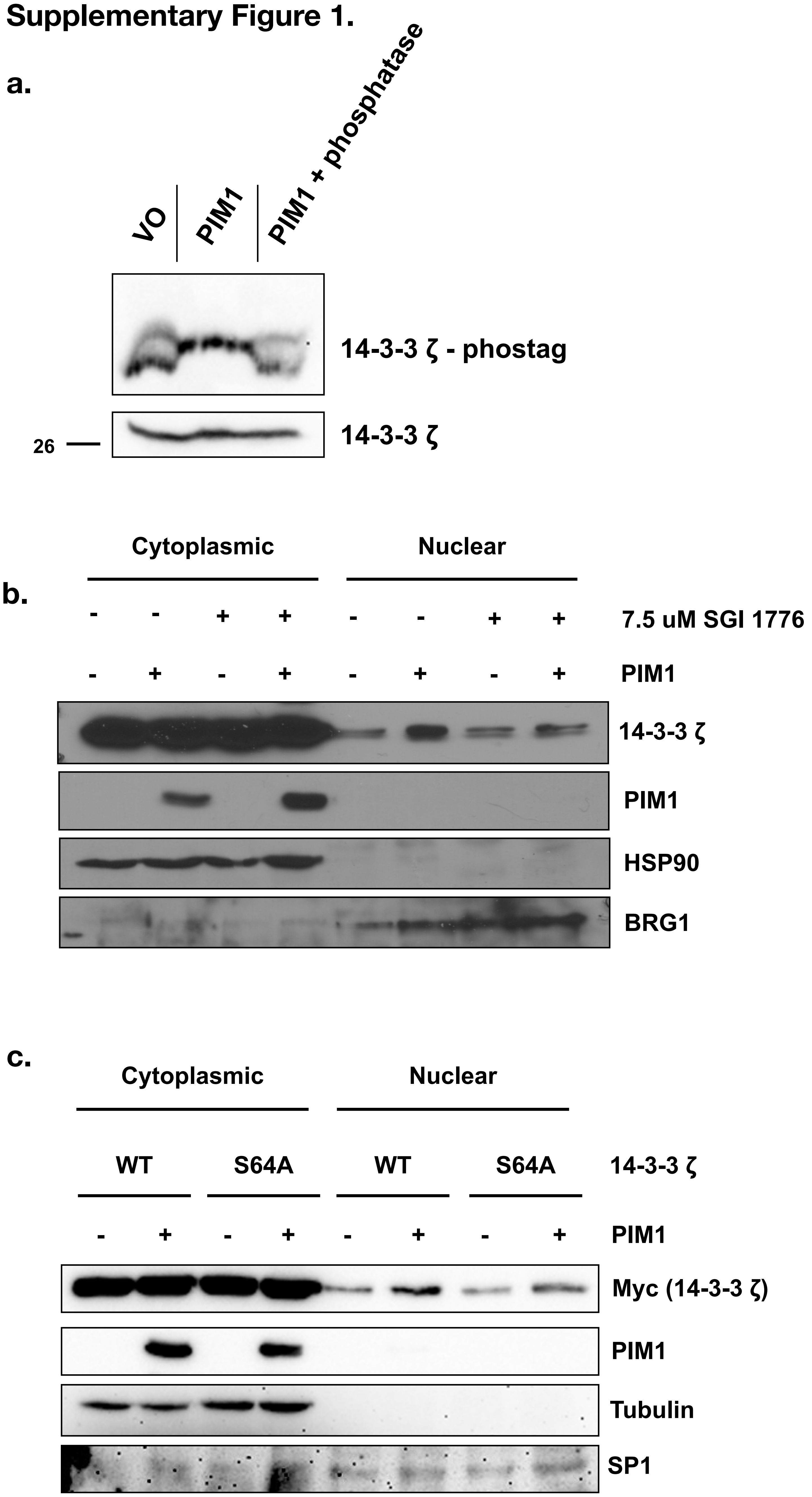

Supplementary Figure 1. PIM1 phosphorylation of 14-3-3 ζ increases its translocation to the nucleus. **A)** PIM1 over-expression in 293 cells results in 14-3-3 ζ phosphorylation on phos-tag gel, which is reversible with phosphatase inhibitor. **B)** PIM1 over-expression in LNCaP cells increases endogenous 14-3-3 ζ translocation to the nucleus, which is reversed by treatment with the PIM1 inhibitor SGI-1776. C**)** Overexpressed WT 14-3-3 ζ increases nuclear translocation in the presence of PIM1 over-expression, which is less true for 14-3-3 ζ S64A.

Supplementary Figure 2.

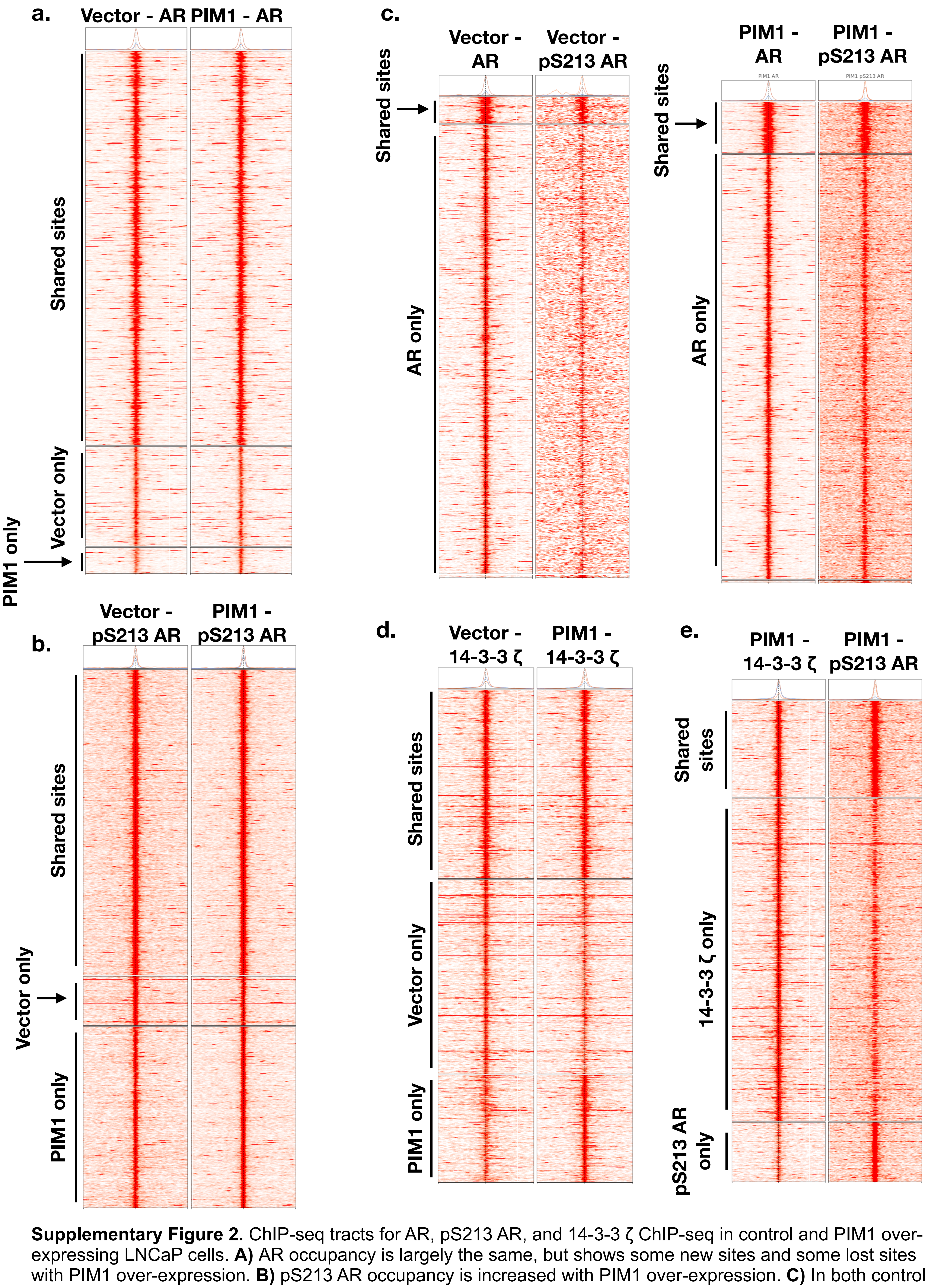

Supplementary Figure 2. ChIP-seq tracts for AR, pS213 AR, and 14-3-3 ζ ChIP-seq in control and PIM1 over-expressing LNCaP cells. **A)** AR occupancy is largely the same, but shows some new sites and some lost sites with PIM1 over-expression. **B)** pS213 AR occupancy is increased with PIM1 over-expression. **C)** In both control and PIM1 over-expressing cells, pS213 AR sites almost completely overlap with AR sites. **D)** 14-3-3 ζ occupancy shifts with PIM1 over-expression. **E)** The majority of AR pS213 occupied sites are also occupied by 14-3-3 ζ.

Supplementary Figure 3.

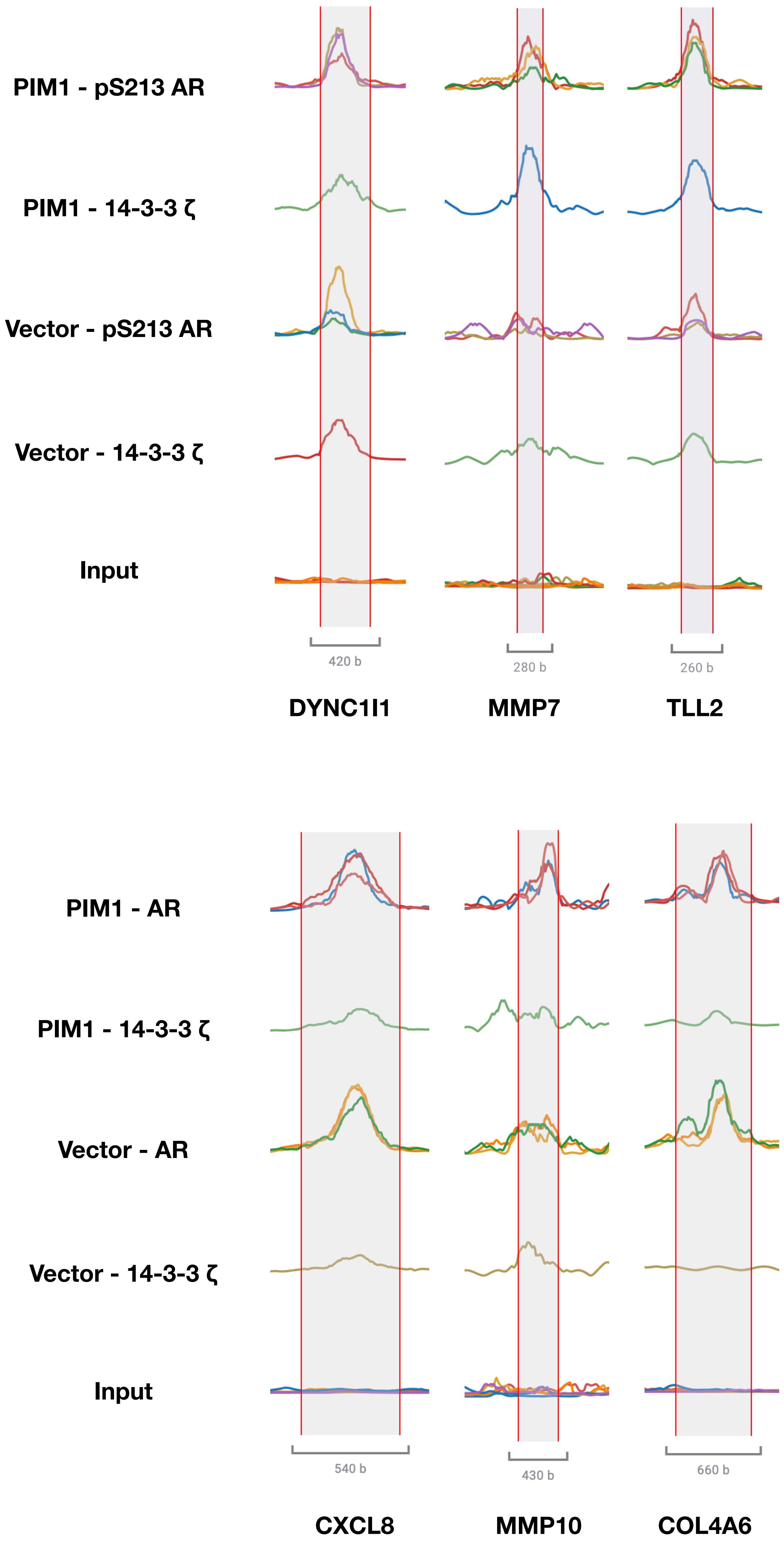

Supplementary Figure 3. ChIP-seq tracts for DYNC1I1, MMP7, and TLL2 showing pS213 AR and 14-3-3 ζ occupancy, and CXCL8, MMP10, and COL4A6 showing total AR and 14-3-3 ζ occupancy.

Supplementary Figure 4.

a.

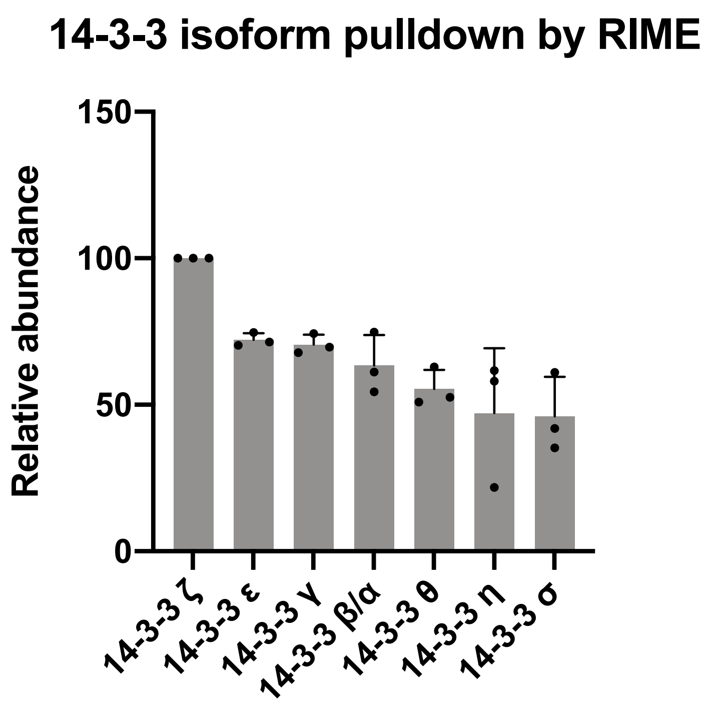

b.

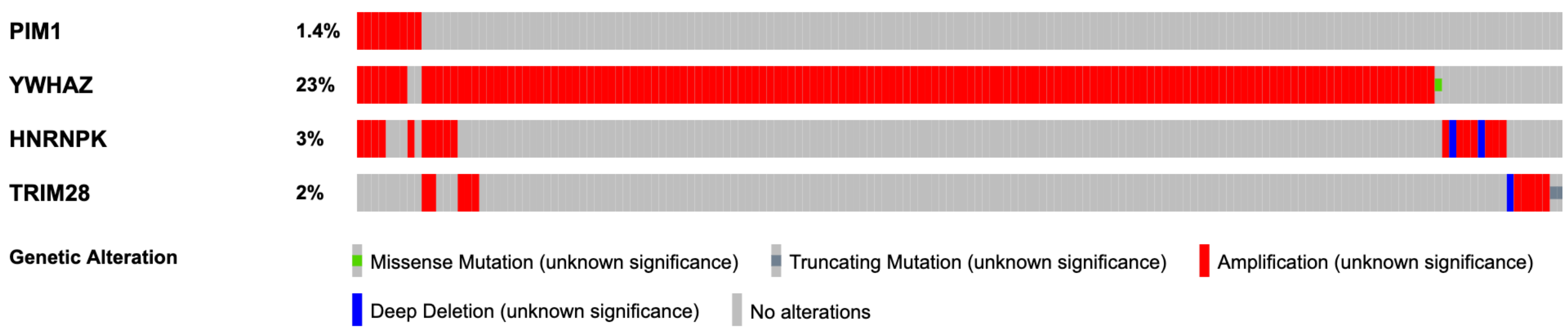

c.

A	B	Neither	A Not B	B Not A	Both	Log2 Odds Ratio	p-Value	q-Value	Tendency
**PIM1**	**HNRNPK**	**632**	**4**	**14**	**5**	**>3**	**<0.001**	**<0.001**	**Co-occurrence**
**PIM1**	**YWHAZ**	**503**	**2**	**143**	**7**	**>3**	**<0.001**	**0.002**	**Co-occurrence**
**YWHAZ**	**HNRNPK**	**495**	**141**	**10**	**9**	**1.66**	**0.015**	**0.03**	**Co-occurrence**
**HNRNPK**	**TRIM28**	**625**	**17**	**11**	**2**	**2.741**	**0.051**	**0.077**	**Co-occurrence**
YWHAZ	TRIM28	497	145	8	5	1.099	0.154	0.185	Co-occurrence
PIM1	TRIM28	633	9	13	0	<-3	0.834	0.834	Mutual exclusivity

Bold = statistically significant relationship

Supplementary Figure 4. RIME data identifies HNRNPK and TRIM28 as cofactors interacting with both AR and 14-3-3 ζ. **A)** 14-3-3 isoform distribution for 14-3-3 ζ RIME pulldown, showing that 14-3-3 ζ is the predominant isoform immunoprecipitated. **B)** PIM1, YWHAZ (14-3-3 ζ), HNRNPK and TRIM28 amplification in metastatic castration resistant prostate cancer. Image used from cBioPortal (citations in text). **C)** PIM1 amplification statistically significantly correlates with YHWAZ (14-3-3 ζ) amplification as well as HNRNPK amplification, while neither YHWAZ or PIM1 significantly correlate with TRIM28 amplification. Table used from cBioPortal (citations in text).

Supplementary Figure 5.

1a

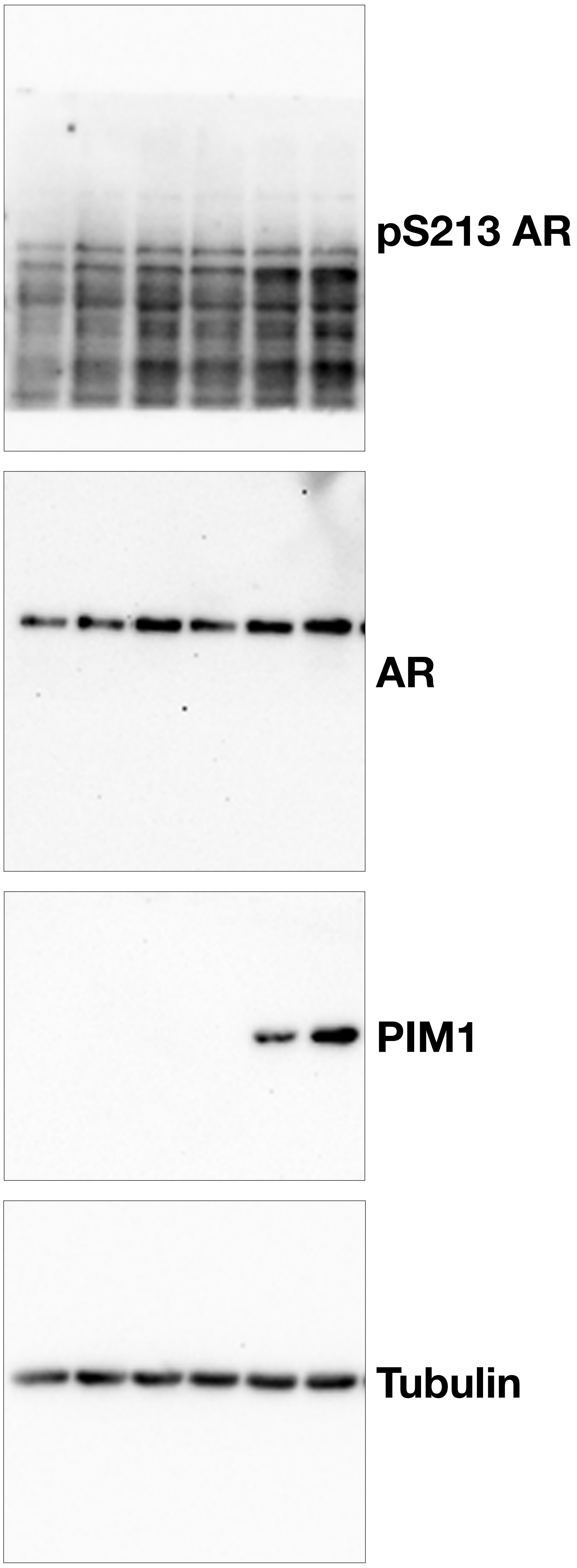

1e

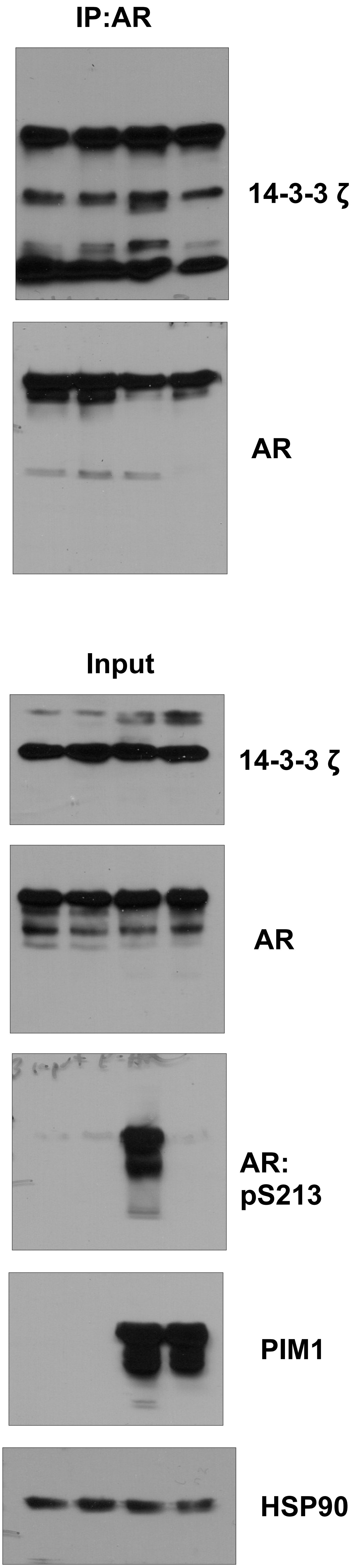

Supplementary Figure 5. Uncropped Western blots.

1f

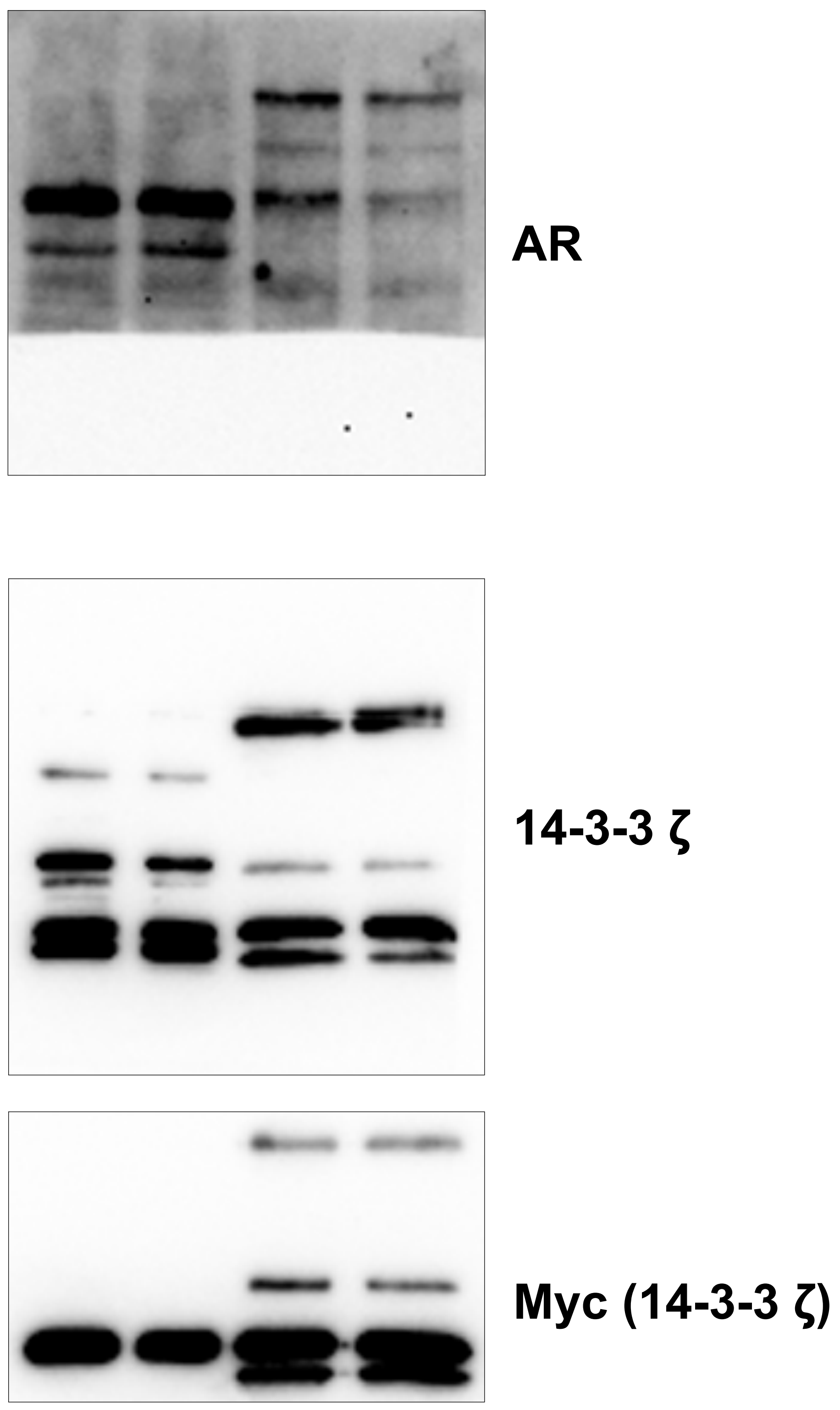

8c

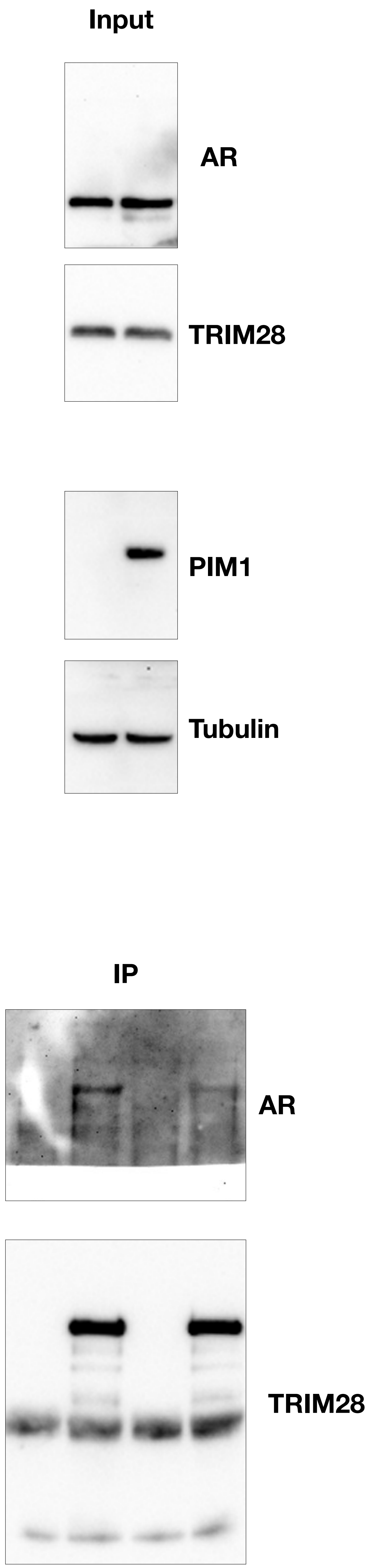

Supplement: Supplementary file 2 — Supplementary Information [file 42003_2021_2723_MOESM2_ESM.pdf]
