# Supplementary material for: PIM1 phosphorylation of the androgen receptor and 14-3-3 ζ regulates gene transcription in prostate cancer
Source: Commun Biol. 2021 Oct 25;4:1221. doi: 10.1038/s42003-021-02723-9 (PMC8546101; doi:10.1038/s42003-021-02723-9)
Supplement: Supplementary file 3 — Description of Additional Supplementary Files [file 42003_2021_2723_MOESM3_ESM.pdf]

Description of Additional Supplementary Files

File name: Supplementary Data 1

Description: AR (Table 1) and 14-3-3 zeta (Table 2) interacting proteins identified by mass spec in 3 independent experiments, and the spectra counts. Table 3 shows proteins that overlap between AR and 14-3-3 zeta. Table 4 shows the amount of AR pulled down by 14-3-3 zeta and *vice versa*.

File name: Supplementary Data 2

Description: Primary source data for the figures.
