# Supplementary material for: PIM1 phosphorylation of the androgen receptor and 14-3-3 ζ regulates gene transcription in prostate cancer
Source: Commun Biol. 2021 Oct 25;4:1221. doi: 10.1038/s42003-021-02723-9 (PMC8546101; doi:10.1038/s42003-021-02723-9)
Supplement: Supplementary file 6 — Reporting Summary [file 42003_2021_2723_MOESM6_ESM.pdf]

nature research

Corresponding author(s): Michael Garabedian and Susan Logan

Last updated by author(s): Aug 10, 2021

# Reporting Summary

Nature Research wishes to improve the reproducibility of the work that we publish. This form provides structure for consistency and transparency in reporting. For further information on Nature Research policies, see our Editorial Policies and the Editorial Policy Checklist.

## Statistics

For all statistical analyses, confirm that the following items are present in the figure legend, table legend, main text, or Methods section.

| n/a | Confirmed | |
|---|---|---|
| ☐ | ☒ | The exact sample size ($n$) for each experimental group/condition, given as a discrete number and unit of measurement |
| ☐ | ☒ | A statement on whether measurements were taken from distinct samples or whether the same sample was measured repeatedly |
| ☐ | ☒ | The statistical test(s) used AND whether they are one- or two-sided<br>*Only common tests should be described solely by name; describe more complex techniques in the Methods section.* |
| ☒ | ☐ | A description of all covariates tested |
| ☒ | ☐ | A description of any assumptions or corrections, such as tests of normality and adjustment for multiple comparisons |
| ☐ | ☒ | A full description of the statistical parameters including central tendency (e.g. means) or other basic estimates (e.g. regression coefficient) AND variation (e.g. standard deviation) or associated estimates of uncertainty (e.g. confidence intervals) |
| ☐ | ☒ | For null hypothesis testing, the test statistic (e.g. $F$, $t$, $r$) with confidence intervals, effect sizes, degrees of freedom and $P$ value noted<br>*Give P values as exact values whenever suitable.* |
| ☒ | ☐ | For Bayesian analysis, information on the choice of priors and Markov chain Monte Carlo settings |
| ☒ | ☐ | For hierarchical and complex designs, identification of the appropriate level for tests and full reporting of outcomes |
| ☒ | ☐ | Estimates of effect sizes (e.g. Cohen's $d$, Pearson's $r$), indicating how they were calculated |

*Our web collection on statistics for biologists contains articles on many of the points above.*

## Software and code

Policy information about availability of computer code

| | |
|---|---|
| Data collection | qPCR data was collected using Applied Biosystems Quantstudio 6 Flex Real-Time PCR System. Mass spec peptides were analyzed by LC-MS on Orbitrap Fusion Lumos mass spectrometer coupled with Dionex Ultimate 3000 UHPLC |
| Data analysis | Statistical testing was completed using Prism 8 for Mac OS X, Version 8.4.3. Sequencing experiments were analyzed using ROSALIND® (https://rosalind.onramp.bio/), details are in the methods. For mass spectometry data, Peptides identification and label-free quantitation was done in Proteome Discoverer 2.1 or MaxQuant 1.6. Search engines, Sequest and Andromeda respectively, were supplied with protein database consisting of human or mouse proteome downloaded from UniProt (www.uniprot.org) combined with a set of known protein contaminants (supplied with MaxQuant). |

For manuscripts utilizing custom algorithms or software that are central to the research but not yet described in published literature, software must be made available to editors and reviewers. We strongly encourage code deposition in a community repository (e.g. GitHub). See the Nature Research guidelines for submitting code & software for further information.

## Data

Policy information about availability of data

All manuscripts must include a data availability statement. This statement should provide the following information, where applicable:

- Accession codes, unique identifiers, or web links for publicly available datasets
- A list of figures that have associated raw data
- A description of any restrictions on data availability

All data generated or analyzed during this study are included in this published article (and its supplementary information files). The mass spectrometry proteomics data have been deposited to the ProteomeXchange Consortium via the PRIDE partner repository with the dataset identifiers PXD023623 and PXD023634 94

(associated with Fig. 1C and Fig. 7). The sequencing data have been deposited into Gene Expression Omnibus database (GEO) under reference series GSE181226 (GSE181224 for ChIP-seq data, GSE181225 for RNA-seq data) (associated with Fig. 2 and Fig. 3).

# Field-specific reporting

Please select the one below that is the best fit for your research. If you are not sure, read the appropriate sections before making your selection.

☒ Life sciences ☐ Behavioural & social sciences ☐ Ecological, evolutionary & environmental sciences

For a reference copy of the document with all sections, see nature.com/documents/nr-reporting-summary-flat.pdf

# Life sciences study design

All studies must disclose on these points even when the disclosure is negative.

| | |
|---|---|
| Sample size | *Describe how sample size was determined, detailing any statistical methods used to predetermine sample size OR if no sample-size calculation was performed, describe how sample sizes were chosen and provide a rationale for why these sample sizes are sufficient.* |
| Data exclusions | No data were excluded from the analyses. |
| Replication | All experiments were reproduced 2-3 times and had high reproducibility. |
| Randomization | Samples were randomly allocated into experimental groups. |
| Blinding | Blinding was not possible due to labeling of experimental materials, but is not necessary since the methods used are objective quantifications, including qPCR cycle numbers. |

# Reporting for specific materials, systems and methods

We require information from authors about some types of materials, experimental systems and methods used in many studies. Here, indicate whether each material, system or method listed is relevant to your study. If you are not sure if a list item applies to your research, read the appropriate section before selecting a response.

## Materials & experimental systems

| n/a | Involved in the study | |
|---|---|---|
| ☐ | ☒ | Antibodies |
| ☐ | ☒ | Eukaryotic cell lines |
| ☒ | ☐ | Palaeontology and archaeology |
| ☒ | ☐ | Animals and other organisms |
| ☒ | ☐ | Human research participants |
| ☒ | ☐ | Clinical data |
| ☒ | ☐ | Dual use research of concern |

## Methods

| n/a | Involved in the study | |
|---|---|---|
| ☐ | ☒ | ChIP-seq |
| ☒ | ☐ | Flow cytometry |
| ☒ | ☐ | MRI-based neuroimaging |

## Antibodies

| | |
|---|---|
| Antibodies used | Commercial antibodies used were as follows: PIM1 (sc-13513; Santa Cruz Biotechnology), 14-3-3 ζ (Western blot and IP for Western blots: sc- 293415; Santa Cruz Biotechnology, ChIP seq and RIME: ab51129; Abcam), AR (Western blot and IP for Western blots: sc-7305; Santa Cruz Biotechnology, ChIP-seq and RIME: blend of sc-7305; Santa Cruz Biotechnology and #5153; Cell Signaling), hnRNPK (ab39975, Abcam), TRIM28 (ab10483, Abcam), HSP90 (610418, BD Biosciences), Tubulin (MMS-489P, Covance), Myc-tag (#2276, Cell Signaling), SP1 (PIPA529165, Fisher Scientific), and BRG1 (ab4081, Abcam). Immunoprecipitations for Western blot were carried out using Protein A/G PLUS-agarose beads (SCBT). pS213 AR antibody was generated by our lab as described in Taneja et al 2005. |
| Validation | All antibodies for Western blot and IP were used for applications which are validated on the manufacturer's website. For ChIP experiments, AR and 14-3-3 ζ antibodies used in IP-mass spec experiments under the same conditions as ChIP seq indicate that AR and 14-3-3 ζ are the predominant proteins identified, validating the specificity of the antibodies. For pS213 AR, the ChIP-seq data indicates overlap with the total AR antibody, and an increase in signal with the presence of PIM1, corresponding to an increase in abundance of pS213 AR in these cells. This antibody is further validated in Taneja et al 2005. hnRNPK and TRIM28 antibodies were previously used for ChIP as cited in the article file. |

# Eukaryotic cell lines

Policy information about cell lines

| | |
|---|---|
| Cell line source(s) | Cell lines used in this study were purchased from ATCC, with the exception of LAPC4 cells, which were a gift from Dr. R. Reiter (University of California, Los Angeles). |
| Authentication | Cell lines were not authenticated. |
| Mycoplasma contamination | Cell lines tested negative for mycoplasma testing. |
| Commonly misidentified lines (See ICLAC register) | No commonly misidentified lines were used in this study. |

# ChIP-seq

## Data deposition

☒ Confirm that both raw and final processed data have been deposited in a public database such as GEO.

☒ Confirm that you have deposited or provided access to graph files (e.g. BED files) for the called peaks.

| | |
|---|---|
| Data access links<br>*May remain private before publication.* | The sequencing data have been deposited into Gene Expression Omnibus database (GEO) under reference series GSE181226 (GSE181224 for ChIP-seq data). |

Files in database submission

| | | | | |
|---|---|---|---|---|
| GSM5492899 | PIM1 10 Rb IgG | Jul 16, 2022 | approved | BIGWIG |
| GSM5492900 | PIM1 20 Rb IgG 1 | Jul 16, 2022 | approved | BIGWIG |
| GSM5492901 | PIM1 20 Rb IgG 2 | Jul 16, 2022 | approved | BIGWIG |
| GSM5492902 | PIM1 20 Rb IgG 3 | Jul 16, 2022 | approved | BIGWIG |
| GSM5492903 | PIM1 Abcam 14-3-3 z | Jul 16, 2022 | approved | BIGWIG |
| GSM5492904 | PIM1 AR 1 | Jul 16, 2022 | approved | BIGWIG |
| GSM5492905 | PIM1 AR 2 | Jul 16, 2022 | approved | BIGWIG |
| GSM5492906 | PIM1 AR 3 | Jul 16, 2022 | approved | BIGWIG |
| GSM5492907 | PIM1 Input 1 | Jul 16, 2022 | approved | BIGWIG |
| GSM5492908 | PIM1 Input 2 | Jul 16, 2022 | approved | BIGWIG |
| GSM5492909 | PIM1 Input 3 | Jul 16, 2022 | approved | BIGWIG |
| GSM5492910 | PIM1 MsRb IgG 1 | Jul 16, 2022 | approved | BIGWIG |
| GSM5492911 | PIM1 MsRb IgG 2 | Jul 16, 2022 | approved | BIGWIG |
| GSM5492912 | PIM1 MsRb IgG 3 | Jul 16, 2022 | approved | BIGWIG |
| GSM5492913 | PIM1 pS213 AR 1 | Jul 16, 2022 | approved | BIGWIG |
| GSM5492914 | PIM1 pS213 AR 2 | Jul 16, 2022 | approved | BIGWIG |
| GSM5492915 | PIM1 pS213 AR 3 | Jul 16, 2022 | approved | BIGWIG |
| GSM5492916 | Vector 10 Rb IgG | Jul 16, 2022 | approved | BIGWIG |
| GSM5492917 | Vector 20 Rb IgG 1 | Jul 16, 2022 | approved | BIGWIG |
| GSM5492918 | Vector 20 Rb IgG 2 | Jul 16, 2022 | approved | BIGWIG |
| GSM5492919 | Vector 20 Rb IgG 3 | Jul 16, 2022 | approved | BIGWIG |
| GSM5492920 | Vector Abcam 14-3-3 z | Jul 16, 2022 | approved | BIGWIG |
| GSM5492921 | Vector AR 1 | Jul 16, 2022 | approved | BIGWIG |
| GSM5492922 | Vector AR 2 | Jul 16, 2022 | approved | BIGWIG |
| GSM5492923 | Vector AR 3 | Jul 16, 2022 | approved | BIGWIG |
| GSM5492924 | Vector Input 1 | Jul 16, 2022 | approved | BIGWIG |
| GSM5492925 | Vector Input 2 | Jul 16, 2022 | approved | BIGWIG |
| GSM5492926 | Vector Input 3 | Jul 16, 2022 | approved | BIGWIG |
| GSM5492927 | Vector MsRb IgG 1 | Jul 16, 2022 | approved | BIGWIG |
| GSM5492928 | Vector MsRb IgG 2 | Jul 16, 2022 | approved | BIGWIG |
| GSM5492929 | Vector MsRb IgG 3 | Jul 16, 2022 | approved | BIGWIG |
| GSM5492930 | Vector pS213 AR 1 | Jul 16, 2022 | approved | BIGWIG |
| GSM5492931 | Vector pS213 AR 2 | Jul 16, 2022 | approved | BIGWIG |
| GSM5492932 | Vector pS213 AR 3 | Jul 16, 2022 | approved | BIGWIG |

| | |
|---|---|
| Genome browser session (e.g. UCSC) | *Provide a link to an anonymized genome browser session for "Initial submission" and "Revised version" documents only, to enable peer review. Write "no longer applicable" for "Final submission" documents.* |

## Methodology

| | |
|---|---|
| Replicates | ChIP-seq for AR and pS213 AR were completed for 3 replicates of each sample with good agreement between replicates. 14-3-3 ζ ChIP-seq was completed with 1 replicate per sample, but peaks of interest were validated using ChIP-PCR. |
| Sequencing depth | All samples had between 13 and 22 million paired-end (PE50) sequencing reads. |
| Antibodies | AR: blend of sc-7305; Santa Cruz Biotechnology and #5153; Cell Signaling. pS213 AR: generated by our lab as described in Taneja et al 2005. 14-3-3 ζ: ab51129; Abcam. |

| | |
|---|---|
| Peak calling parameters | Peaks were called using MACS26 (with input control background subtracted). Peaks were compared with IgG controls to eliminate any nonspecific peaks. |
| Data quality | *Describe the methods used to ensure data quality in full detail, including how many peaks are at FDR 5% and above 5-fold enrichment.* |
| Software | Data was analyzed by ROSALIND® (https://rosalind.onramp.bio/), with a HyperScale architecture developed by OnRamp BioInformatics, Inc. (San Diego, CA). Reads were trimmed using cutadapt. Quality scores were assessed using FastQC. Reads were aligned to the Homo sapiens genome build hg19 using bowtie2. Per-sample quality assessment plots were generated with HOMER and Mosaics. Peaks were called using MACS26 (with input/IgG controls background subtracted). Peak overlaps and differential binding were calculated using the DiffBind R library. Differential binding was calculated at gene promoter sites. Read distribution percentages, identity heatmaps, and FRiP plots were generated as part of the QC step using ChIPQC R library and HOMER. |
